# Core-shell microstructured nanocomposites for synergistic adjustment of environmental temperature and humidity

**DOI:** 10.1038/srep36974

**Published:** 2016-11-15

**Authors:** Haiquan Zhang, Yanping Yuan, Nan Zhang, Qingrong Sun, Xiaoling Cao

**Affiliations:** 1 School of Mechanical Engineering, Southwest Jiaotong University, 610031, Chengdu, China; 2 School of Civil Engineering and Architecture, ChongQing University of Science and Technology, Chongqing 401331, People’s Republic of China

## Abstract

The adjustment of temperature and humidity is of great importance in a variety of fields. Composites that can perform both functions are prepared by mixing phase change materials (PCMs) with hygroscopic materials. However, the contact area between the adsorbent and humid air is inevitably decreased in such structures, which reduces the number of mass transfer channels for water vapor. An approach entailing the increase in the mass ratio of the adsorbent is presented here to improve the adsorption capacity. A core-shell CuSO_4_/polyethylene glycol (PEG) nanomaterial was developed to satisfy the conflicting requirements of temperature control and dehumidification. The results show that the equilibrium adsorption capacity of the PEG coating layer was enhanced by a factor of 188 compared with that of the pure PEG powder. The coating layer easily concentrates vapor, providing better adsorption properties for the composite. Furthermore, the volume modification of the CuSO_4_ matrix was reduced by 80% by the PEG coated layer, a factor that increases the stability of the composite. For the phase change process, the crystallization temperature of the coating layer was adjusted between 37.2 and 46.3 °C by interfacial tension. The core-shell CuSO_4_/PEG composite reported here provides a new general approach for the simultaneous control of temperature and humidity.

Adjusting environmental temperature and humidity is of critical importance in a variety of situations, be it general engineering, national defense, social productivity, or simple day-to-day activities^1^,^2^,^3^,^4^,^5^. Integrated temperature and humidity control for the above application areas can be achieved via composites obtained by mixing phase change materials (PCMs) with hygroscopic materials. Such composites have many advantages, such as high dehumidification efficiency and zero noise, and also have a smaller impact on air pollution compared to alternative technologies. Furthermore, these absorbent materials can achieve long-term, stable management of environmental temperature and humidity by utilizing cheap energy sources such as solar energy, geothermal energy, and factory waste heat^6^,^7^.

PCMs^8^,^9^,^10^, or “latent heat-storage materials”, are classified into two major categories: inorganic and organic materials. Inorganic PCMs^8^,^11^, including hydrated salts, molten salts, and alloys, have high thermal conductivities and energy densities. However, strong corrosion, high undercooling, and poor chemical stability hinder their practical usage. In contrast, organic PCMs have attracted significant attention for their high thermal storage densities and excellent chemical and thermal stabilities^11^,^12^. Indeed, polyethylene glycols (PEGs)^13^,^14^,^15^ of grades ranging from 400 to 1,000,000 have been investigated as PCMs using both computational and experimental methods. In order to enhance their efficiency, metal foams, polymer networks, microcapsules, and porous matrices^13^,^15^ have been developed as supports for shape-stabilized PCMs. In addition, surface modification of such support matrices has also been researched extensively. For example, Dong *et al*.^16^ reported the grafting of –OH, –NH_2_, and –CH_3_ functional terminal groups onto the surface of silica gel particles to regulate the crystallization and stabilization behavior of PEG. It has also been found that incorporation of materials with high thermal conductivities, such as activated carbon, graphite, and metals, can enhance the thermal conductivity of PCMs. The study by Ji *et al*.^17^ can be considered as an example, and the results showed that the embedding of ultrathin graphite foams even at a low density of 1.2 vol.% could increase the thermal conductivity of PCMs by a factor of 18.

At present, practical applications of composites used for the control of room temperature and humidity face many hurdles. In these composites, the contact area of the hygroscopic material with wet air is inevitably decreased by the PCM, which reduces the adsorption performance. For example, diatomite composites reach absorption equilibrium only after 48 h^18^. Composites using sepiolite clay as a matrix material display poor adsorption performance^19^, with water uptake limited to 1.2–2.1 g/100 g. One of the most common approaches for improving the adsorption capacities of such composites is enhancing the mass ratio of the adsorbent. Unfortunately, this method negatively affects the thermal storage performance. Chen *et al*.^20^ reported that the enthalpy of fusion can be as low as 19 J/g for some composites that utilized the above strategy.

PEG polymers display a high supercooling (D-value between the melting temperature and actual crystallization temperature) value of 20 °C^13^,^21^,^22^. Common approaches toward adjusting the phase transition temperature include multicomponent communion^23^,^24^,^25^,^26^ and microencapsulation nanotechnology^27^,^28^,^29^,^30^. The latter can regulate the crystallization temperature, and does not affect the melting point. In general, the melting point of a microencapsulated PCM is lower than that of the bulk PCM, but the variation of the crystallization temperature is no more than 3 °C.

In this study, a core-shell CuSO_4_/PEG (CS/PEG) nanocomposite was fabricated by *in situ* liquid deposition. The synthetic method involved obtaining ultrafine nanocrystals of the CuSO_4_ matrix through a drying process. During this process, alcohol and PEG molecules act as surfactants and reduce the surface energy of the nanocrystals, preventing further grain growth. Both the crystalline and molecular structures of the PEG coating layer were modified by the interfacial interactions between the PCM molecules and the support matrix surface. The conflicting requirements of controlling the temperature and dehumidification were managed by optimizing the synergistic parameters of the endothermal-hygroscopic materials, as will be describe in detail below. As a result, the water uptake of the PEG shell was improved by a factor of 188 relative to that of pure PEG. To achieve this, the coating layer concentrates the vapor during the adsorption process, improving the adsorption properties of the overall composite. Meanwhile, the PEG coated layer reduced the volume change of the CuSO_4_ matrix, greatly improving the stability of the microstructure of the composite. Finally, the variation in the crystallization temperature reached 9 °C due to surface tension modification, while the melting point of the PEG remained unchanged. This superior performance observed in this work indicates that composite materials with large internal interfacial areas could have significant potential for applications in adjusting room temperature and humidity.

## Results and Discussion

### Synthesis of the CuSO_4_ nanomaterials

**Figure 1:**
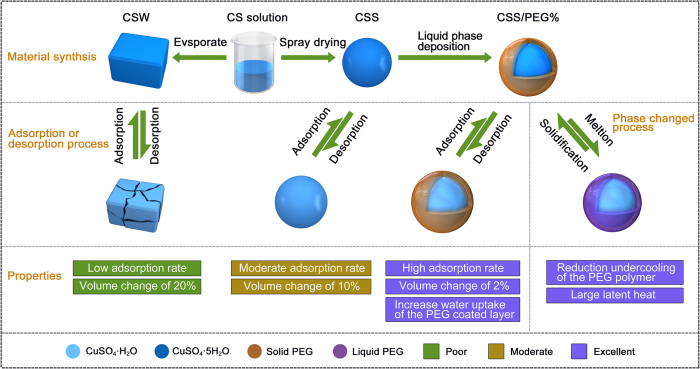
Scheme of the synthetic approach, absorption/desorption characteristics, and phase-change properties of the pristine CuSO_4_ and CS/PEG *X*% materials.

**Figure 2:**
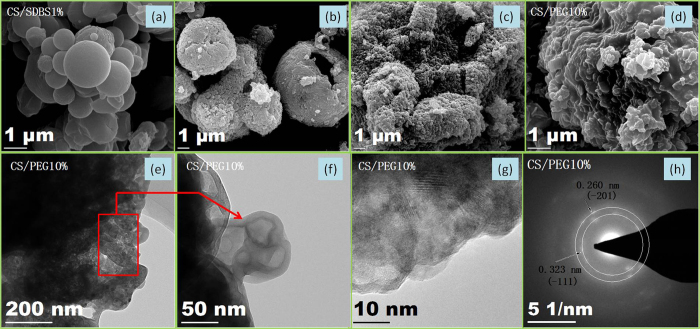
SEM (**a–d**) HRTEM (**f,g**) and SAED (**h**) images of all samples. (**a**) CS/SDBS 1% composite; (**b**) CuSO_4_ sample obtained by drying the CS/SDBS 1% material in air; (**c**) the CS/SDBS 1% sample after dispersion in anhydrous ethanol and synthesis by heating the mixture; and (**d–h**) the CS/PEG 10%.

**Figure 3:**
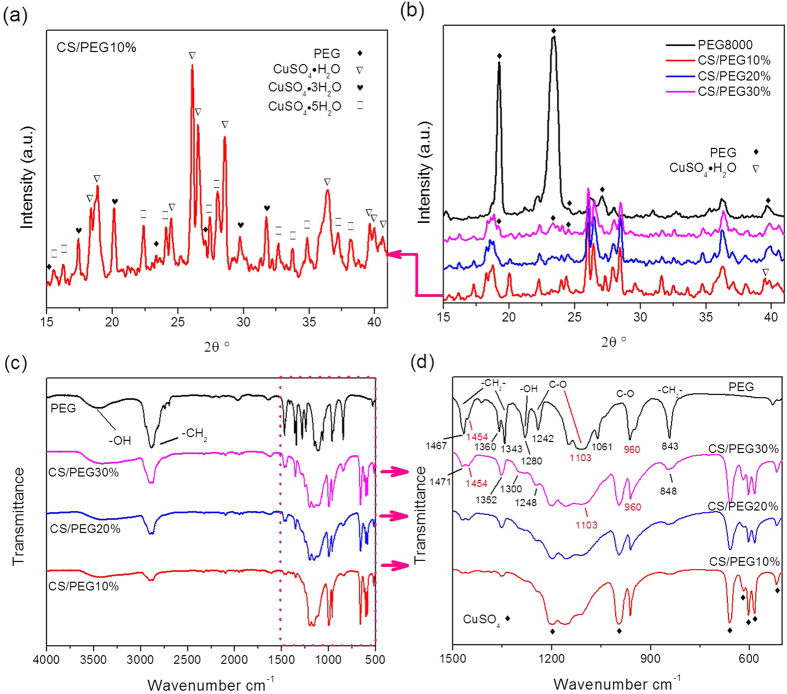
XRD patterns (**a,b**) and FT-IR curves (**c,d**) of the pure PEG powder and CS/PEG X% (X = 10, 20, and 30).

The synthesis methodologies and endothermal-hygroscopic properties are displayed in Fig. 1. Optical microscopy (OM) images indicate that the CSW powder is composed of irregularly shaped particles with a maximum size of a few hundred micrometers (Fig. 1a). As seen in Fig. 1b, the pristine CS/SDBS 1% sample consists of uniform spheres with a size of 2 μm. Clearly, the CuSO_4_ nanomatrix can be easily obtained via the spray-drying technique, and in order to synthesize a nanomaterial with excellent morphological structure, the CuSO_4_ and SDBS concentrations were optimized. SEM images of the resultant CuSO_4_ nanomaterials are presented in Fig. 2. Solid spheres 0.1–2 μm in size are observed with 1 wt.% SDBS dispersed in a 0.2 g cm^−3^ CuSO_4_ solution, as shown in Fig. 2a. When the mass ratio of the SDBS exceeded 3%, the synthesized samples consisted of irregularly shaped large particles. To measure the composition and crystal structure of the CS/SDBS 1% sample, X-ray diffraction (XRD) analysis was employed, with the results presented in Fig. 3. The most intense diffraction peaks appear at 16.2, 18.7, 22.2, and 23.9°, which are indexed respectively as the (−110), (0–11), (−1–21), and (−121) planes of the chalcanthite structure (JCPDS card no. 11–0646). Based on these results, the pristine CS/SDBS 1% nanospheres were used as the matrix material for the endothermal-hygroscopic composites.

### Physical characteristics of the CS/PEG endothermal-hygroscopic composites

**Figure 4:**
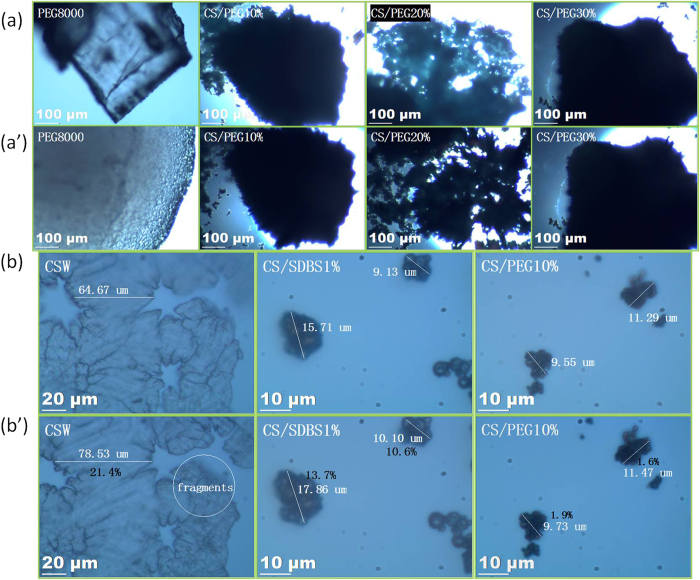
OM images of pure PEG, CSW, CS/SDBS 1%, and CS/PEG X% (X = 10, 20, and 30) composites. All samples are shown (**a**) before and (**a′**) after thermal treatment; (**b**) before and (**b′**) after adsorption of water vapor.

Crystalline CuSO_4_·5H_2_O experiences significant changes in both volume and crystal structure during the desorption process. The microstructure of pure CuSO_4_ exhibits obvious fragmentation during the adsorption and desorption cycles. As shown in Fig. 2c, unstable hollow spheres are prepared by drying the CS/SDBS 1% sample in air. These spheres accumulate into evenly distributed primary nanoparticles with sizes of 0.5–2 μm. In order to prevent further growth of these primary grains in high-temperature environments, the CS/SDBS 1% powder was placed in ethanol, so that the medium acts as a surfactant. The nanomaterials obtained in this manner display fairly uniform particle sizes between 50 and 200 nm, as shown in Fig. 2c. This demonstrates that the ethanol molecules reduce the surface energy of the nanomatrix. In order to further decrease the surface energy of these CuSO_4_ particles, an *in situ* liquid deposition technique was applied to prepare the CS/PEG *X*% (X = 10, 20, and 30) composites, as described earlier in the experimental section. These composites, each assembled from a large number of the earlier nanoparticles, show solid ball-like structures several micrometers in size (Figs 2d and 4). To analyze the morphology of these nanoparticles, transmission electron microscopy (TEM) images were obtained and selected-area electron diffraction (SAED) analysis was performed. According to Fig. 2(e–g), a core-shell structure is found in the CS/PEG 10% composite. The CuSO_4_ core material displays a uniform nanoparticle size between 5 and 50 nm. The PEG coating layer has a thickness of ~10 nm. From Fig. 2h, it is evident that the matrix material is CuSO_4_·H_2_O.

Adsorption experiments on pure PEG and the CS/PEG *X*% (X = 10, 20, and 30) composites were performed at 25 °C and at a relative humidity of 50%. The XRD patterns of all samples with crystal hydrate structures are given in Fig. 3. The crystal diffraction peaks for CuSO_4_·H_2_O, CuSO_4_·3H_2_O, and CuSO_4_·5H_2_O^31^,^32^ are found in the core-shell structured CS/PEG 10% composite, as seen in Fig. 3a. The absorption equations that establish a dynamic equilibrium between these species are given below:


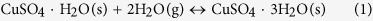



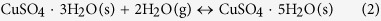


In addition, the crystal diffraction peaks of PEG8000 are observed at 15.2, 27.1, and 36.2° in the XRD patterns of the above composite. This demonstrates that PEG is deposited on the surfaces of the CuSO_4_ nanoparticles as a separate physical phase. In Fig. 3b, pristine PEG crystals exhibit strong diffraction peaks at 19.2 and 23.4°. However, these peaks cannot be observed clearly at the same positions in the CS/PEG 10% and CS/PEG 20% samples. For the CS/PEG 30% composite, weak diffraction peaks appear at 19.2 and 23.4°, while strong peaks are found at 24.5 and 27.0°. This demonstrates that in these structures, the preferential face of crystal growth of the PEG coating layer is different from that of pure PEG. Furthermore, a diffraction peak appears at 39.6° in the pattern of the CS/PEG 10%, but not in CS/PEG 20% or CS/PEG 30%. This illustrates that the crystal structure of the PEG layer is different in the three composites.

In order to demonstrate the modification of the surface functional groups, the samples were analyzed by FT-IR as shown in Fig. 3c,d. The IR absorption peaks of the C-O and –OH groups appear at 1242, 1103, 960, and 1280 cm^−1^ for pure PEG. However, in the composites, the sharp peaks of C–O are measured at 1248, 1103, and 960 cm^−1^, while the absorption peaks at 1300 cm^−1^ are attributed to the –OH bonds. In addition, the peak of the interim –CH_2_– groups in the CS/PEG 10%, 20%, and 30% composites change in location from 843, 1343, and 1467 cm^−1^ to 848, 1352, and 1471 cm^−1^, with the latter values observed for the higher concentrations. In summary, some absorption peaks observed in the composites appear in the spectrum of pure PEG with a slight shift, indicating that the vibration modes of the C-O, –OH, and –CH_2_– functional groups are modified by interaction with the supporting matrix.

### Stability tests of the CS/PEG endothermal-hygroscopic composites

**Figure 5:**
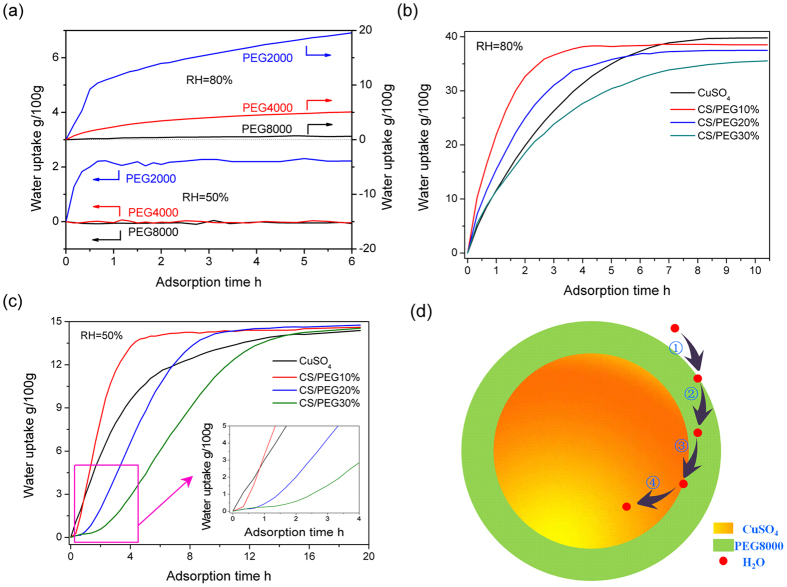
Absorption profiles of all samples. The water uptake of the PEG shell is calculated as follows: 
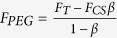
(3); where *F*_T_ and *F*_CS_ are the equilibrium adsorption capacity of the composite and pristine CuSO_4_ nanomaterial, respectively (g/100 g), and *β* is the mass ratio of the CuSO_4_ matrix in the composite (%). Detailed results are given in (Supporting Information Table 1).

Figure 5 depicts digital photographs of the CSW, CS/SDBS 1%, and CS/PEG *X*% (X = 10, 20, and 30) materials. No liquid is observed in any of the samples at 125 °C, indicating that all composites are shape-stabilized PCMs. To verify this observation, OM imaging is performed before and after the thermal treatment, as shown in Fig. 4a,a′, respectively. The PEG is present as elongated particles before the thermal treatment. After heating for 2 h at 75 °C, the PEG polymer shrinks to form spherical particles as a result of surface tension, yielding the structure depicted in Fig. 4a′. However, in the CS/PEG *X*% (X = 10, 20, and 30) composites, the microscale topology remains unchanged after exposure to high temperatures. This suggests that PEG can be restrained by the matrix to resist surface tension-induced shape changes.

As a further test of stability, the pure CSW, CS/SDBS 1% inorganic material, and CS/PEG *X*% (X = 10, 20, and 30) composites were placed in a thermostat-humidistat chamber at 25 °C and 60% relative humidity for 12 h. The microscale topology of the above materials was tested before and after the adsorption of water vapor under these conditions, as depicted in Fig. 4b,b′, respectively. The CSW, obtained by evaporating water slowly from CS solutions, displays an obvious cubic dilatation of more than 20%. The volume expansion accompanying adsorption is reduced to 10% for the spherical CS/SDBS 1% sample. For the material coated with PEG, the volume change is less than 2%. Therefore, the coated CS/PEG materials exhibits the most stable microstructure among the nanomaterials synthesized in this work.

**Figure 6:**
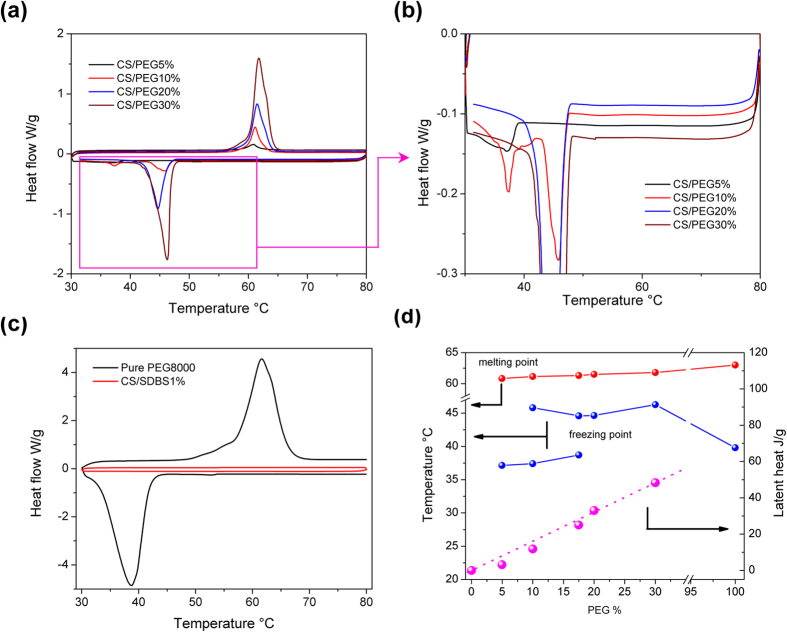
DSC analysis of (**a**) CS/SDBS 1%, (**b**) PEG8000 and (**c**) composites; and (**d**) the phase change temperatures and latent heats of the composites with 5, 10, 17.5, 20, and 30 wt.% PEG8000.

Thermogravimetric analysis (TGA) of the pristine PEG8000, CuSO_4_·H_2_O, CS/SDBS 1%, and CS/PEG *X*% (X = 10, 20, and 30) composites confirms that the PEG molecules are stably contained on the surface of the CuSO_4_ matrix, as seen in Fig. 6. The mass loss of CuSO_4_·H_2_O during TGA analysis can be divided into three processes, as shown in Fig. 6a, and corresponds to the syntheses of CuSO_4_·3H_2_O (40–60 °C), CuSO_4_·H_2_O (60–85 °C), and CuSO_4_ (200–270 °C). The decomposition temperature of PEG8000 exceeds 350 °C, but that of the CS/PEG composites is less than 175 °C. TGA also reveals that the CuSO_4_ matrix does modify the microstructure of the PEG coating layer, corroborating the XRD and FT-IR results in Fig. 3. Moreover, the water adsorbed onto the PEG coating layer is removed fully at 70–115 °C. This data suggests a drying temperature of 115–125 °C for all composites.

### Endothermal-hygroscopic characteristics of the CS/PEG composites

Adsorption curves of all samples were obtained at room temperature, and are shown in Fig. 5. It is known that, with increasing molecular weight of a PEG polymer, its water uptake capability decreases (see Fig. 5a). The equilibrium adsorption capacity of the PEG8000 is almost 0 g/100 g at the low relative humidity of 50%. At 80% humidity, the water uptake of PEG8000 remains below 0.6 g/100 g. In contrast, the water uptake of the pure CS/SDBS 1% powder was found to be 19.9, 31.4, and 37.5 g/100 g for adsorption times of 2, 4, and 6 h (Fig. 5b), reaching 50.5%, 78.9%, and 94.2%, respectively, of the equilibrium adsorption capacity of 39.8 g/100 g. Further improvement is seen for the core-shell structured CS/PEG 10% and CS/PEG 20% samples, whose adsorption capacities are 32.7 and 25.0 g/100 g, respectively, after a 2 h period. Clearly, the initial adsorption rates of the CS/PEG 10% and CS/PEG 20% composites are higher than that of pristine CuSO_4_ during the first two hours.

The equilibrium adsorption capacities attributed to the CuSO_4_ nanomatrix are equal to 35.8, 31.8, and 27.9 g/100 g in the CS/PEG *X*% (X = 10, 20, and 30) composites, respectively. In conjunction with the earlier results, this represents net decreases of 2.7, 5.7, and 7.7 g/100 g, respectively, in the composites. As such, the PEG coating layer is clearly involved in the adsorption process of the CuSO_4_, since its water uptake can reach 27 and 15.8 g/100 g at the relative humidity of 80% and 50%, respectively, as shown in (Supporting Information Table 1). These values are equal to 44 and 188 times the capacity of pure PEG. The adsorption property of the PEG coating layer is clearly enhanced when its crystal structure (see XRD data, Fig. 3b) and functional groups (see IR spectra, Fig. 3d) are modified by the CuSO_4_ matrix. At a relative humidity of 50%, the CS/PEG 10% and CS/PEG 20% composites reach adsorption equilibrium in 5 and 10 h, respectively, as shown in Fig. 5c. This is a significant improvement over the uncoated material, where the equilibration time is over 20 h.

Additionally, the respective water uptake rates of the CS/PEG 10%, 20%, and 30% composites approach 0 for the first 20, 40, and 90 min under a relative humidity of 50%. According to Fig. 5(a–c), the adsorption processes in these composites can be divided into four parts: (1) adsorption by the PEG coating; (2) diffusion of water molecules into the PEG bulk material; (3) aggregation of water molecules at the PEG/matrix interface; and (4) diffusion of water molecules into the matrix. Our previous studies^33^,^34^ have shown that organic polyvinylpyrrolidone (PVP) can adsorb water vapor to form high-moisture regions even at low relative humidity. Furthermore, addition of CaCl_2_ as a chemical adsorbent enhanced the diffusion rate of water from such high-moisture regions rather than from air, enhancing the apparent rate of adsorption for the composite material. Similar high-moisture regions are seen on the PEG/matrix interface of the current work. If the concentration of water molecules in these regions exceeds that of air, the adsorption rate is improved for the composite, and vice versa. According to Fick’s first law, the concentration is inversely proportional to the diffusion distance, which is equal to the coating thickness in this case. Consequently, the adsorption rate of CS/PEG 10% is greater than that of the uncoated sample, and that of CS/PEG 30% is over.

Figure 6 shows the differential scanning calorimetry (DSC) analysis of the pure CuSO_4_, PEG8000, and CS/PEG composites. The bulk PEG displays a melting peak at 61.6 °C. The melting points of the CS/PEG composites are between 60.8 and 61.8 °C. This indicates that the modification of the crystal structure and functional groups does not significantly affect the endothermic process. During the exothermic process, the peak temperature of the unmixed powder is ~38.8 °C. However, this value increases to 44.7 and 46.3 °C for the CS/PEG 20% and CS/PEG 30% composites, which are net enhancements of 5.9 and 7.5 °C, respectively. When the PEG content is reduced to 17.5%, double exothermic peaks are observed at 44.6 and 38.7 °C. The solidification point of the composite with 5 wt.% PEG is even lower to 37.2 °C. In summary, the recrystallization temperature of the PEG coating layer can be adjusted by the surface tension interactions between the PEG molecules and the CuSO_4_ support in the range of 37.2 to 46.3 °C. The underlying principle of this mechanism is only partially understood, and further studies on this phenomenon are ongoing.

In Table 1, The endothermal-hygroscopic properties of the CS/PEG composites from this work are compared with those of other reported composites. As mentioned earlier, the hygroscopic and thermal energy storage requirements conflict with each other. Conventional methods enhance the water uptake or the heat storage by increasing the relative content of the adsorbent or PCM, respectively. In this light, our proposal of utilizing the interfacial effect of a nanomatrix-structured adsorbent shows promise. The enthalpy of fusion in these structures increases from 0 J/g for pure CuSO_4_ to 48.3 J/g for CS/PEG 30%, and the D-value (4.2 g/100 g, 10.6 wt%) of water uptake remains nearly constant. The core-shell microstructured composite therefore presents a potential architecture for endothermal-hygroscopic materials for use in applications such as energy-storing wallboards, plastering materials, and dehumidification systems.

## Conclusions

Desiccant/PCM composites are green functional materials where thermal energy storage and release capabilities are combined with the ability to control humidity levels. Such material could have extensive applications. However, in such composites, the addition of the PCM inevitably decreases the contact area of the hygroscopic material with wet air, thereby severely reducing the adsorption performance. Previous methods for enhancing adsorption capacity have all entailed the sacrifice of thermal energy storage density. In this work, we successfully developed a straightforward methodology to prepare microspheres of CuSO_4_/PEG composites with core-shell structures by combining a spray drying technique with *in situ* liquid deposition technology. The particle size of the CuSO_4_ matrix is limited to be between 5 and 50 nm, thus providing abundant internal interfaces in the composite. The crystallization behavior of the PEG is significantly altered after being coated on the CuSO_4_ support matrix, presumably due to the high-energy interfaces. As a result, the water uptake capability of the PEG layer can be increased by up to 188 times relative to that of pure bulk PEG. The coating layer increases the effective concentration of water vapor during the adsorption process, allowing the adsorption properties of the composite to exceed that of pristine CuSO_4_. Meanwhile, the CuSO_4_ exhibits obvious volume and crystal structure changes during adsorption and desorption cycling. However, growing a layer of PEG several nanometers in thickness on the surface of the CuSO_4_ matrix can reduce the above volume change by at least 80%. This greatly improves the microstructural stability of the composite. For the phase-change process, the crystallization temperature of the PEG coating layer is adjusted between 37.2 and 46.3 °C by the interfacial tension, and its melting point remains unchanged. This composite structure therefore represents a new technology for the management of the phase transition temperature. The water uptake rate decreases from 39.8 g/100 g in the pristine CuSO_4_ matrix to 35.6 g/100 g in the core-shell CS/PEG 30% composite. However, the heat storage capacity is increased significantly from 0 J/g to 48.3 J/g. Thus, the CS/PEG composite provides a novel architecture for the simultaneous regulation of the ambient temperature and humidity, with wide applicability in engineering applications.

## Methods

### Preparation of the CuSO_4_ and CS/PEG nanomaterials

All experimental chemicals were of analytical grade and used as-received without further purification. The synthetic methodology and the endothermal-hygroscopic properties of interest are described in Fig. 1. In the first step, spherical CuSO_4_·5H_2_O nanomaterials were synthesized through a typical spray-drying technique using CuSO_4_ solutions of 0.1, 0.2, and 0.3 g cm^−3^, and are named as CS 10%, CS 20%, and CS 30%, respectively. As a control, blue CuSO_4_·5H_2_O powder was prepared by slow evaporation of the solutions. This control material was labeled CSW. Subsequently, sodium dodecyl benzene sulfonate (SDBS) was added with stirring to the aqueous solutions of CS nanomaterials at a concentration of 0.2 g cm^−3^, over a period of 2 h at 60 °C. The bulk samples obtained by spray-drying were labeled CS/SDBS *X*% (where X = 1 to 3).

The PEG coating layer was deposited on the surface of the CuSO_4_ nanomatrix by liquid-phase deposition. The CS/SDBS 1% powder and the PEG8000 polymer were mixed at PEG mass ratios of 5, 10, 17.5, 20, and 30 wt.%. These mixtures were dispersed in ethanol with stirring for 12 h at 80 °C. The resulting core-shell composites were labeled CS/PEG *X*% (X = 5, 10, 17.5, 20, and 30).

### Material characterization

The morphologies of the samples were observed using an optical microscope (OLYMPUS CX31), a field-emission scanning electron microscope (FE-SEM, Hitachi, S3400N), and a high-resolution transmission electron microscope (HR-TEM, JEOL, JEM-100CX). X-ray diffraction measurements were performed using Cu Kα (λ = 0.154056 nm) radiation with a step size of 0.03° in the 2θ range of 15° to 40°. The molecular structures were identified by Fourier-transform infrared spectrometry (FT-IR, Nicolet 5700, USA) in the wavenumber range from 500 to 4000 cm^−1^. For TGA, the Q500 instrument was used with a heating rate of 10 °C min^−1^ from 30 to 500 °C in air. The adsorption equilibrium between water vapor and the composite was studied in a thermostat-humidistat chamber with uncertainties of ±0.5 °C for temperature and ±5% for humidity. The melting temperatures and latent heats of the composites were obtained using a differential scanning calorimeter (TAQ20 USA) at 5 °C min^−1^ under a constant stream of argon at a flow rate of 50 mL min^−1^.

## Additional Information

**How to cite this article**: Zhang, H. *et al*. Core-shell microstructured nanocomposites for synergistic adjustment of environmental temperature and humidity. *Sci. Rep.* **6**, 36974; doi: 10.1038/srep36974 (2016).

**Publisher’s note**: Springer Nature remains neutral with regard to jurisdictional claims in published maps and institutional affiliations.

## Supplementary Material

Supplementary Information

## Figures and Tables

**Table 1:** Comparison of the endothermal-hygroscopic properties of the CS/PEG composites synthesized in this work with those of composites reported in recent literature.

Composite material	Freezing point (°C)	Latent heat (J/g)	Water uptake (g/100 g)	Reference
PCM (5.6 wt%)/SiO_2_/diatomite	26.8	8.3	10.2	[18]
PCM (11.5 wt%)/SiO_2_/diatomite	26.9	17.1	10.3	
PCM (19.1 wt%)/SiO_2_/diatomite	27.0	28.2	10.5	
PCM (63.4 wt%)/diatomite	26.7	89.6	—	[20]
PCM (12.9 wt%)/diatomite	26.7	18.4	2.5	
Sepiolite (25.46 wt%)/Na_2_SO_4_·10H_2_O	—	122.7	2.2	[19]
Sepiolite (61.66 wt%)/paraffin	—	145.7	1.7	
Sepiolite (65.83 wt%)/lauryl alcohol	—	79.02	1.5	
CS/PEG 5%	37.2	8.1	—	present
CS/PEG 10%	37.2	11.8	38.5	
	44.1			
CS/PEG 17.5%	38.7	25	—	
	41.6			
CS/PEG 20%	44.7	33	37.5	
CS/PEG 30%	46.3	48.3	35.6	
