# Supplementary material for: Core-shell microstructured nanocomposites for synergistic adjustment of environmental temperature and humidity
Source: Sci Rep. 2016 Nov 15;6:36974. doi: 10.1038/srep36974 (PMC5109540; doi:10.1038/srep36974)
Supplement: Supplementary Information [file srep36974-s1.pdf]

# Core-shell microstructured nanocomposite for synergistic adjustment of environmental temperature and humidity

Haiquan Zhang, Yanping Yuan*, Nan Zhang, Qingrong Sun and Xiaoling Cao

School of Mechanical Engineering, Southwest Jiaotong University, 610031 Chengdu, China

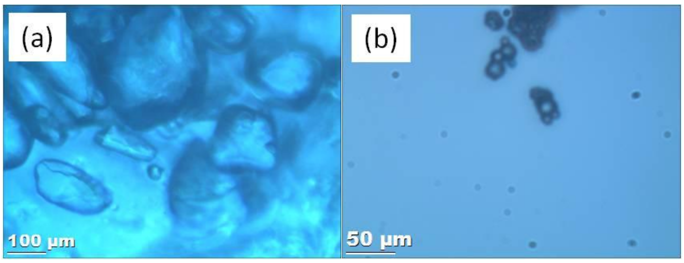


Figs. 1 Optical microscope images of pristine $CuSO_4$ powders prepared by the evaporation (a, b) and spray drying technique (c, d).

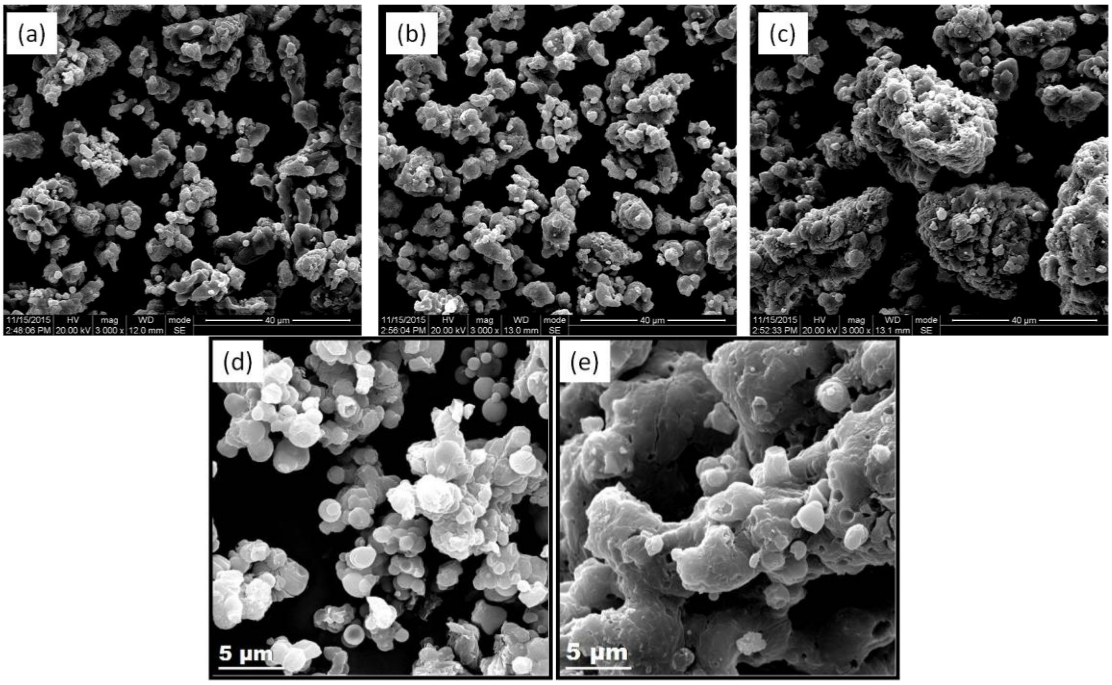


Figs. 2 SEM images of the $CuSO_4$ nanoparticles with the concentration of 0.1 (a), 0.2 (b) and 0.3 (c) g/ml via the spray drying methode; the $CuSO_4$ samples obtained under the SDBS concentration of 1% (d) and 3% (e).

* Corresponding author. Tel.: +86 28 87634937; fax: +86 28 87634937.
*E-mail address*: ypyuan@home. swjtu.edu.cn.(Y. Yuan)

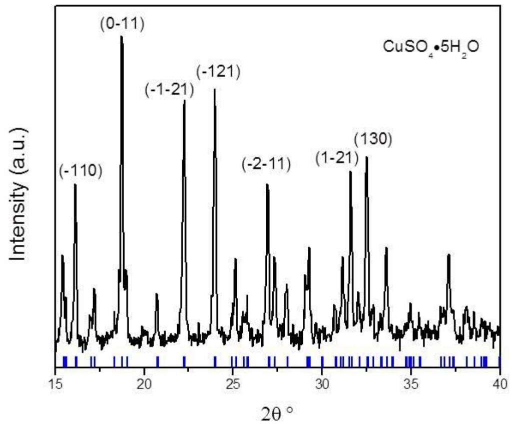

Figs. 3  XRD pattern of the CS/SDBS1% nanomaterial.

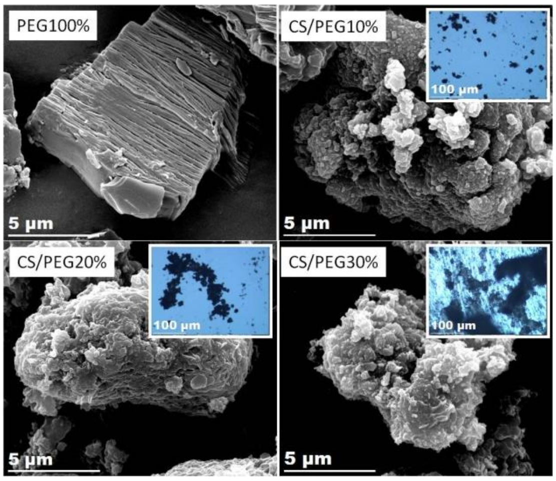

Figs. 4 SEM and optical microscope images of the PEG and CS/PEGx% (X=10, 20 and 30)

Figs. 4 exhibits the SEM and optical microscope images of the PEG and CS/PEGx% (X=10, 20 and 30). Pure PEG8000 polymer with laminated microstructure comprises many large particles with the size of 5 μm. The nano-based PEG film can be synthesized by depositing polymer on the matrix surface for the CS/PEG10%. When the mass ratio of the PEG organic increases, the agglomerated secondary particles present greater diameter as shown in optical microscope images.

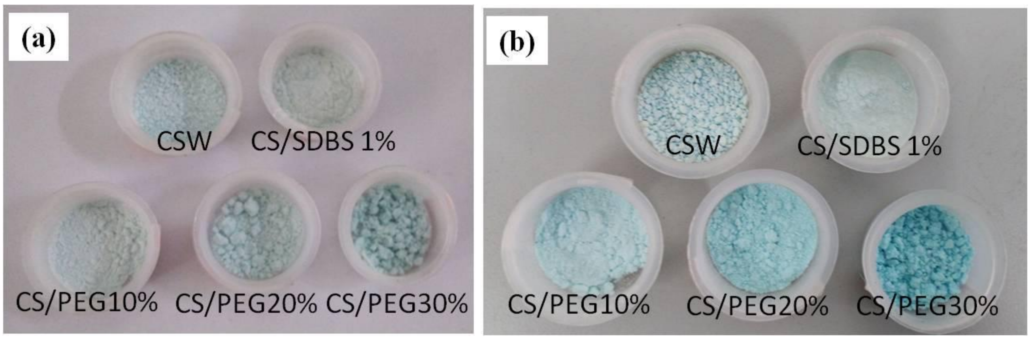


Figs. 5 Digital photos of the pure CSW, CS/SDBS 1% materials and CS/PEG *X*% (X=10, 20 and 30) composites. (a) All samples were placed in an oven at 125 ℃ for 2 h; (b) All samples had adsorbed vapor at 25 ℃ and RH=50%.

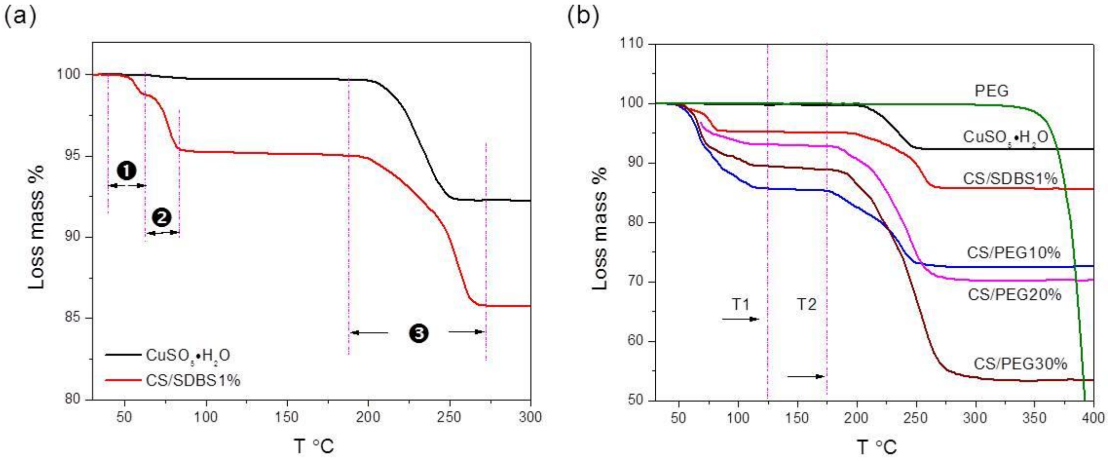


Figs. 6 TG curves of the pristine CS1 and CS/SDBS1% smaples,

and CS/PEGx% (X=10, 20 and 30) composites. Therein,

①: $CuSO_4 \cdot 5H_2O\ (s) \rightarrow CuSO_4 \cdot 3H_2O\ (s) + 2H_2O\ (g)$ ;

②: $CuSO_4 \cdot 3H_2O\ (s) \rightarrow CuSO_4 \cdot H_2O\ (s) + 2H_2O\ (g)$ ;

③: $CuSO_4 \cdot H_2O\ (s) \rightarrow CuSO_4 (s) + H_2O\ (g)$ ;

T1: Drying temperature; and T2: Decomposition temperature.

Table 1 Equilibrium adsorption capacity of the all samples during the relative humidity of 80%(a) and 50% (b). Therein, (A) Total equilibrium adsorption capacity of the all samples; (B) Water uptake of the $CuSO_4$ matrix for all the samples; (C) Water uptake of PEG for all the composite; and (D) Equilibrium water uptake of the PEG coated layer.

(a) During the 80% relative humidity

| | Water uptake (g/100g) | | | |
|---|---|---|---|---|
| | A | B | C | D |
| $CuSO_4$ | 39.8 | 39.8 | 0 | |
| CS/PEG10% | 38.5 | 35.8 | 2.7 | 27 |
| CS/PEG20% | 37.5 | 31.8 | 5.7 | 28 |
| CS/PEG30% | 35.6 | 27.9 | 7.7 | 26 |
| PEG bulk | 0.59 | 0 | 0.59 | 0.6 |

The PEG coated layer displays high water uptake of 27 g/100g during the relative humidity of 80%, and is equal to 44 times of the pure PEG powder.

(b) Under the relative humidity of 50% condition

| | Water uptake (g/100g) | | | |
|---|---|---|---|---|
| | A | B | C | D |
| $CuSO_4$ | 14.4 | 14.4 | 0 | |
| CS/PEG10% | 14.6 | 13.0 | 1.6 | 16 |
| CS/PEG20% | 14.8 | 11.5 | 3.3 | 16.5 |
| CS/PEG30% | 14.6 | 10.1 | 4.5 | 15 |
| PEG bulk | 0.084 | 0 | 0.084 | 0.084 |

The water uptake of the coating layer can reach 15.8 g/100g, and is more than 188 times that of the pure PEG.
